# Genome-Wide Identification of Epigenetic Hotspots Potentially Related to Cardiovascular Risk in Adult Women after a Complicated Pregnancy

**DOI:** 10.1371/journal.pone.0148313

**Published:** 2016-02-12

**Authors:** Cees Oudejans, Ankie Poutsma, Omar Michel, Joyce Mulders, Allerdien Visser, Marie van Dijk, Tessa Nauta, Anouk Bokslag, Walter Paulus, Andreas de Haas, Pieter Koolwijk, Christianne J. M. de Groot

**Affiliations:** 1 Department of Clinical Chemistry and Institute for Cardiovascular Research (ICaR-VU), VU University Medical Center, De Boelelaan 1117, 1081 HV, Amsterdam, The Netherlands; 2 Department of Physiology, VU University Medical Center, De Boelelaan 1117, 1081 HV, Amsterdam, The Netherlands; 3 A-Skin BV, Amsterdam, De Boelelaan 1118, 1081 HZ, Amsterdam, The Netherlands; 4 Department of Obstetrics/Gynaecology, VU University Medical Center, De Boelelaan 1117, 1081 HV, Amsterdam, The Netherlands; Victor Chang Cardiac Research Institute, AUSTRALIA

## Abstract

**Background:**

The physiological demands of pregnancy on the maternal cardiovascular system can catapult women into a metabolic syndrome that predisposes to atherosclerosis in later life. We sought to identify the nature of the epigenomic changes associated with the increased cardiovascular disease (CVD) risk in adult women following pre-eclampsia.

**Findings:**

We assessed the genome wide epigenetic profile by methyl-C sequencing of monozygotic parous twin sister pairs discordant for a severe variant of pre-eclampsia. In the adult twin sisters at risk for CVD as a consequence of a complicated pregnancy, a set of 12 differentially methylated regions with at least 50% difference in methylation percentage and the same directional change was found to be shared between the affected twin sisters and significantly different compared to their unaffected monozygous sisters.

**Conclusion:**

The current epigenetic marker set will permit targeted analysis of differentially methylated regions potentially related to CVD risk in large cohorts of adult women following complicated pregnancies.

## Background

Cardiovascular disease (CVD) is the single leading cause of death for women in developed countries and claims the lives of more women than all forms of cancer combined. All women face the threat of cardiovascular disease. However, symptoms of cardiovascular disease tend to occur about 10 years later in women than in men, while women often have different symptoms of coronary artery diseases than men. Pregnancy allows a unique opportunity to challenge these gender-specific differences at the earliest stage possible. The physiological demands of pregnancy on the maternal cardiovascular system and the need to establish a connection between the maternal and fetal vascular systems can catapult a woman into a metabolic syndrome that predisposes to vascular endothelial dysfunction and atherosclerosis in adult life [1]. In a prospective study of 3,416 women, the association with the calculated 10-year CVD risk based on the Framingham prediction score was odds ratio 1.31 (95% confidence interval, 1.11–1.53) for pre-eclampsia [2].

This 10-year CVD risk differs for the various forms of gestational hypertension. Major differences in the clinical presentation of pre-eclampsia and other hypertensive disorders result from differences in underlying mechanisms, which have varying implications for CVD in later life especially for methods of prevention to be effective. Broadly, two risk groups can be discriminated [2–5]. Women with common pre-existing risk factors of various nature (pre-pregnancy hypertension, diabetes mellitus, obesity and/or dislipidaemia) mostly present with gestational hypertension that is benign and late. Persistence after pregnancy is common while the birth weight of the newborn is normal. In other words, the predisposing subclinical risk already existing prior to pregnancy in these women exacerbates by the challenge of pregnancy. This group is the largest in number with the long term CVD risk being difficult to analyze given the diverse and complex nature of the pre-existing factors. The second group, women that develop (pre)eclampsia and related syndromes (Hemolysis, Elevated Liver enzymes, Low Platelets) (HELLP), has no pre-existing risk factors of this kind. These women present relatively early in pregnancy with multisystem dysfunction with the syndrome starting in the placenta in the first trimester. Consequently, children born to this group of mothers frequently have a low birth weight [2–5].

The latter group provides a unique opportunity to explore the mechanism(s) underlying the maternal risk of CVD after a complicated pregnancy. First, the inverse relationship between maternal risk of CVD mortality and infant birth weight is well recognized [3]. As such, targeting the group with the highest risk and greatest correlation, i.e. representative of the extreme end of the pregnancy complications (HELLP syndrome, being a severe variant of pre-eclampsia), will permit the best discrimination possible between the pathways involved in CVD and those reflecting normal biological variation between and within individuals. Secondly, discordance for pre-eclampsia is the rule rather than the exception in monozygotic parous twin sisters [6]. Consequently, the long-term persistence of CVD risk in discordant twin sisters can only be explained by a resetting of the *epi*genome in the affected twin sister. Long-term genetic memory in the absence of DNA changes is by definition epigenetic. When specific regions of the maternal epigenome are being reset by pre-eclampsia or HELLP with permanent changes in the nature and number of epigenomic marks (mostly 5-methylcytosine), this epigenetic memory can lead to persistent changes in the transcription of specific genes given the location of the epigenomic marks in regulatory regions such as CpG islands. When the set of genes that are permanently have been reset are associated and/or expressed by the cardiovascular system, this will lead to an increased CVD risk [7].

Here we address the question which epigenomic changes are associated with the increased CVD risk in adult women after pre-eclampsia or HELLP by performing genome-wide methyl-C sequencing of monozygotic twin sister pairs discordant for the HELLP syndrome.

## Methods

### Epigenetic risk profiling by whole-genome bisulfite sequencing

Genomic DNA was isolated by affinity-based DNA isolation (Qiagen) from buffy coats of EDTA blood from two monozygous parous twin sister pairs discordant for HELLP (n = 4). The protocol **(S3 File,** protocol) was approved by the medical board of the VU Medical Center Amsterdam (**S1 File,** **NL38972.029.12,** approved on April 24^th^, 2013) and registered as trial (**S2 File**, **NTR5297).** All women gave written informed consent. Women were invited on August 19^th^, 2014, informed consent was signed between August 31th and September 25^th^, 2014. The blood was withdrawn from the twin sisters 2 years after the complicated pregnancy (from September 26^th^ and November 7^th^, 2014). Between the period of the affected pregnancies and the blood withdrawal (DNA isolation and analysis, no other pregnancies occurred that could have influenced our results. The affected sisters presented with de novo hypertension and proteinuria before week 34 and abnormal levels of LDH (> 600 IU/l) and ALAT/ASAT (at least 70 IU/l) with low platelets (< 100 platelets x 10^9^/l). Purified (OD 260/280 1.8–2.0) genomic DNA (5 μg/sample) was fragmented by Covaris shearing (S2/E210) to peak sizes of 250 bp as verified with a DNA 1000 chip on a Agilent 2100 Bioanalyzer, end-repaired using T4 DNA polymerase and Klenow enzyme for 3’ overhang removal and 5’ fill-in reaction, respectively. The product was purified using the QIAquick PCR purification kit, subjected to 3’-ends adenylation, purified using the MinElute PCR purification kit, followed by ligation with methylated adapters in the presence of a molar excess of adapter and purification with the MinElute PCR purification kit. DNA adapter indexes 2, 4, 5 and 6 were used. The methyl-adapter ligated genomic DNA (gDNA) fragments were subjected to a second round of purification using AmPure beads and treated with bisulfite to convert any unmethylated cytosine to thymidine using an adaptation (gel purification replaced by AmPure bead purification) of the EpiTect Bisulphite kit. PCR using Pfu Turbo Cx Hotstart DNA polymerase was applied to enrich for DNA fragments with adapter molecules on both ends and to allow for accurate quantification. Three independent PCR reactions were performed for each sample to maximize yield and diversity. To avoid skewing, PCR cycles were limited to 4. The DNA libraries from each individual sample were pooled, purified with AmPure beads and quantitated on an Agilent Bioanalyzer 2100 using a High Sensitivity DNA chip. Average peak sizes were around 350 bp (100–1000 bp). Whole-genome bisuflite sequencing (WGBS) libraries were diluted to 10 nM in 10 μl. Loading concentration for cBot clustering was 7 pM for each sample in the presence of 5% PhiX. Each lane contained a single sample. In addition, all samples were run in duplicate (paired-end, 200 cycles).

To avoid or reduce mis-mapping events or incorrect methylation calls, the bisulfite-sequence files were subjected to quality- and adapter trimming using TrimGalore. Base calls with a Phred score of 20 or lower, adapter sequences and sequences shorter than 20 bp were removed in addition to one additional base pair from the 3’-end of both reads. The latter step is needed for subsequent alignments of completely overlapping long reads with Bowtie. Trimmed, validated files were checked with FastQC. Bisulfite mapping was performed using Bismark (v0.7.7) [8–10]. Following creation of C>T and G>A versions of the genome (hg19) with indexing, sequences were aligned to the converted genome using default conditions (paired end, directional, Bowtie 1, up to 2 mismatches in the seed region). Statistical testing for differential DNA methylation at single CpGs or larger genomic regions, statistical correction for multiple hypothesis testing and ranking based on statistical significance and effect size was done using SeqMonk (http://www.bioinformatics.babraham.ac.uk/projects/seqmonk). For this, the ‘Bisulphite methylation over features’ pipeline was followed to set reliable observation thresholds followed by comparison between target (affected twins) and control groups (non-affected twins). In short, designing probes with the read position probe generation resulted in about 50 million probes per pair of twin sisters with determination of percentage methylation for all CpGs. Instead of testing for individual CpGs, CpG islands were analyzed using the Bisulfite pipeline over CpG islands to determine the percentage methylation over CpGs. Criteria were a minimum coverage of 2 reads for a position to count and at least 3 different calls per feature. Not well measured CGIs received a quantification of -1. All reads that were not well measured were filtered out (filtered for values and individual probes, both data sets need a value of at least 0 (eliminating the -1 values). Subsequently the statistical filter > ChiSquare > fwd/rev between the two twin sisters (1 vs 2 and 3 vs 4 separately) was applied to identify CGIs that showed a statistical significant overall difference (default parameters and multiple testing correction). Regions with significant changes between affected and non-affected twin pairs were imported as annotation tracks into SeqMonk as well as downloaded as an annotated file with the CGIs and closest gene (2 kb cutoff) with significant differences (Q-score).

### Targeted methyl-specific DNA sequencing

The 777 bp CpG island fragment corresponding to DMR1 (chr1:180922916–1809236920) was used for methyl-specific primer design (Meth-primer). The primers (BSP-1F 5’-GGY GTG GGG TTA GGT AGT ATA-3’ and BSP1-R 5’-ACA TCA CCT TTC AAC AAA ACC-3’) were used for PCR amplication of bisulfite-treated genomic DNA of all twin samples along with normal controls at annealing temperatures of 55, 58, 60 and 62°C followed by sequencing. At the optimal annealing temperature of 62°C, an 80 bp fragment (chr1:180,923,360–180,923,439) containing 16 differentially-methylated C’s in CpG or CHG (where H is A, C or T) context could be scored reliably.

### Phenotypic risk assessment

Epigenetic risk profiling was complemented by phenotypic risk assesment i.e. cardiovascular risk assessment (questionnaire, blood pressure, anthropometrics, blood samples, urine collection and cardiac ultrasound). The questionnaire included social status, medical-, obstetric- and family history, psychological status, lifestyle, use of medication, and contraceptive methods. Blood pressure was measured twice, manually in sitting position. The mean value of two measurements was used. Height was measured with a wall-mounted stadiometer to the nearest 0.5 cm. An analog weight scale was used to measure the body weight in indoor cloths without shoes to the nearest 0.5 kg. Waist and hip circumference was measured on uncovered skin using an inelastic tape measure at the narrowest point of the waist and the widest part of the hips to the nearest 0.5 cm. The venous blood samples were taken after an overnight fast for analysis of glucose, glycated hemoglobin (HbA1c), lipids, lipoproteins, renal function and NT-proBNP. Immediately after waking up, urine was collected at home for microalbuminuria and creatinine. Cardiac ultrasound contained a complete two dimensional echocardiography, Doppler and TDI study using Philips iE33 with S5-1 Matrix transducer [11].

## Results

Two monozygotic twin sister pairs discordant for hypertensive pregnancy disorders (i.e. HELLP syndrome) were analyzed 2 years after pregnancy for epigenetic risk and 6–12 years for phenotypic risk assessment to identify factors associated with cardiovascular risk. Baseline characteristics during pregnancy are shown in **Table 1.** Of each twin sister pair, one sister had the HELLP syndrome during pregnancy, while the other had uncomplicated pregnancies. The hypothesis was tested that the cardiovascular risk profiles induced by a complicated pregnancy with persistence in adult life should be limited to, but shared between the discordant twin sisters and be visible in the phenotype, epigenotype or both.

**Table 1:** Baseline characteristics of index pregnancies.

	Twin A		Twin B	
Hypertensive disorder in pregnancy in history	Yes, HELLP	No	Yes, HELLP	No
Maternal age, years	27	25	34	32
Nulliparity	Yes	No	Yes	No
Diastolic blood pressure at booking, mmHg	80	75	68	Missing
Highest diastolic blood pressure, mmHg	120	75	120	80
Gestational age at delivery, weeks+days	33+2	40+3	37+4	39+1
Birth weight, grams	1560	4125	3420	3650

**Fig 1:**
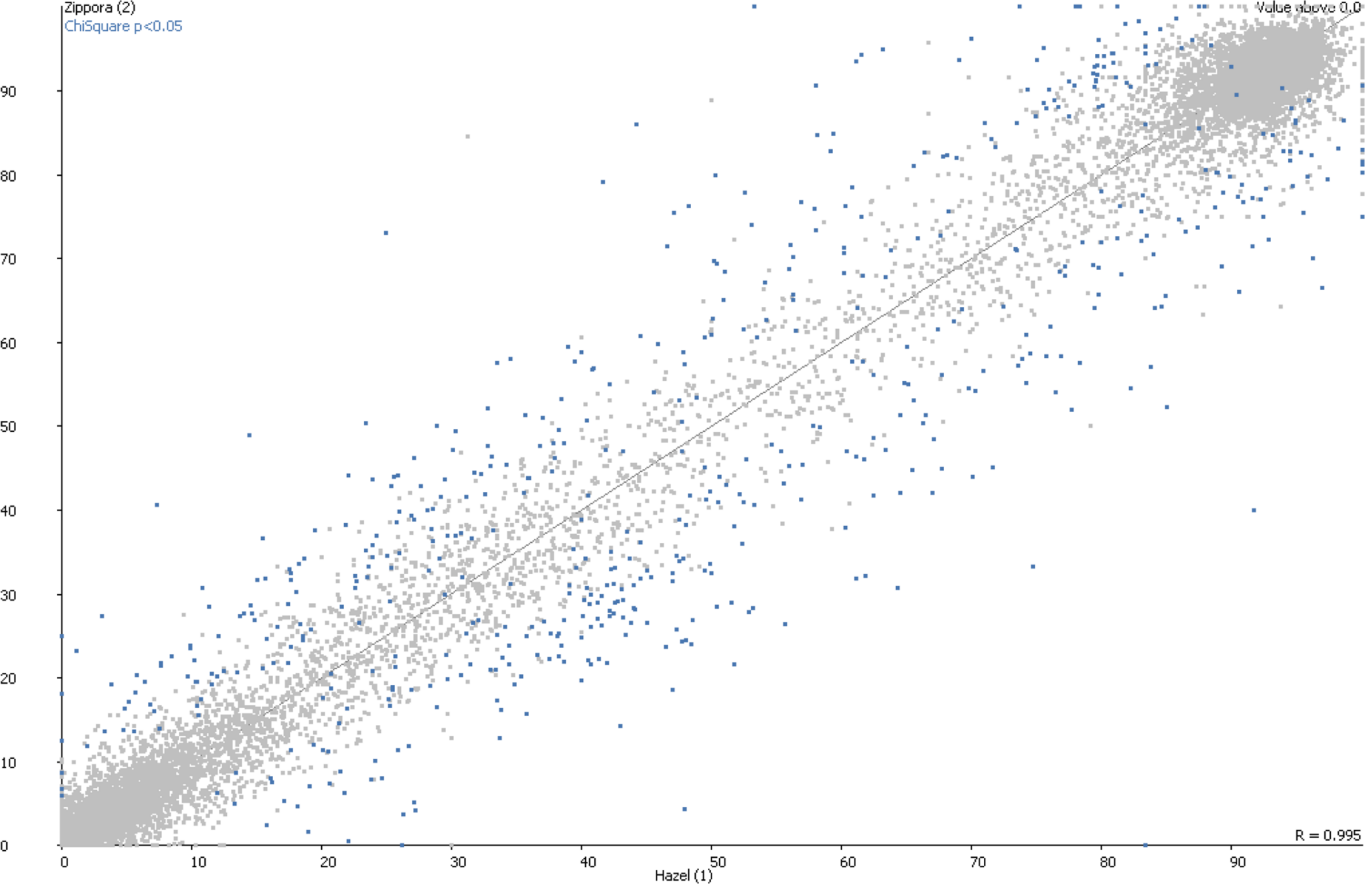
Scatter plot of differentially methylated regions (DMRs) with significant difference between adult monozygotic twins sisters discordant for severe pre-eclampsia and subsequent CVD risk. DMRs were identified by genome-wide methylC-sequencing followed by Bismark analysis.

Of the cardiovascular risk markers (n = 18) tested, no differences were seen between the twin sisters (**Table 2**). Tension, serum markers and echocardiography measurements were all normal. In contrast, out of 22732 epigenetic markers tested, 107 DMRs were changed significantly in all individuals tested (**Fig 1**) and present on all chromosomes except for the Y chromosome as expected. Of these, 12 qualified as differentially methylated regions with a least a 50% difference in methylation percentage, with the same directional change (either up- or downregulation) and shared between and restricted to the affected twin sisters (**Table 3**). Three DMRs were hypomethylated and nine DMRs involved CpG islands with hypermethylation, respectively, i.e likely corresponding to increased and decreased gene expression of the associated genes. Four of these DMRs involved CpG islands without proximity (2 kb cutoff) to a known gene. Three involved annotated genes coding for proteins with yet unknown function. The coding genes of the other DMRs involved transcription factors *(HIVEP3*), cell adhesion proteins (*PCDHA1*), protein kinase (*SRPK3*), proteasome regulatory protein (*PSMD4*), and RNA helicase (*DHX58*).

**Table 2:** Phenotypic outcome in adult life.

	Twin A		Twin B		
Hypertensive disorder in pregnancy in history	Yes	No	Yes	No	
**Measurements and lifestyle**					
Age, years	33	33	44	44	
Systolic blood pressure, mmHg	95	100	110	110	
Diastolic blood pressure, mmHg	58	70	75	76	
BMI, kg/m^2^	22.6	27.7	26.9	27.7	
Waist circumference, cm	73.5	85	85.5	90	
Hip circumference, cm	104	112.5	110.5	114	
Smoking	No	No	No	No	
Medication	No	Levothyroxine	Ciclesonide Salbutamol Desloratadine Psyllium	DesloratadinePantoprazole Movicol	
**Laboratory results**					Ref
Cholesterol, mmol/l	3.9	3.6	5.1	5.2	< 6,5
HDL, mmol/l	1.33	1.31	1.88	1.86	> 0.9
LDL, mmol/l	2.3	2.0	2.9	3.0	< 5.0
Triglyceride, mmol/l	0.6	0.7	0.8	0.8	< 2,0
Fasting glucose, mmol/ml	4.5	4.7	4.6	4.8	< 6.1
HbA1c, mmol/mol	36	33	36	36	23–43
eGFR, mL/min/1.73m2	>90	>90	65	71	>60
NT-proBNP, ng/l	17	23	21	48	<220^1^
Microalbumine in urine, mg/l	4.9	4.3	3.8	< 2.0	< 20
**Echocardiography**				
LVEF, percentage, 2D or 3D analysis	54, 3D	54.4, 3D	60, 2D	60.5, 3D	
LV mass, grams	150	102.4	148.3	139.9	
Diastolic function	Normal	Normal	Normal	Normal	

Abbreviations: BMI, Body Mass Index; Ref. range: reference range in non-pregnant women; HDL, High Density Lipoproteïne; LDL, Low Density Lipoproteïne; HbA1c, Glycated hemoglobin; eGFR, estimated glomerular filtration rate; NT-proBNP, N-terminal brain natriuretic peptide; LVEF, Left ventricular ejection fraction; 2D/3D, 2/3 dimensional; LV, Left ventricular.

**Table 3:** Characteristics of differentially methylated regions with at least 50% difference in methylation percentage, the same directional change and shared between and specific to the affected twin sisters. Boxes 1 and 2: Methylation percentages of CpG islands as analyzed in replicates in 2 discordant monozygotic twins sisters. Box 3: Localization and associated genes (if known) of the 12 regions potentially associated with cardiovascular risk in adult women following pregnancies complicated by pre-eclampsia and/or HELLP.

**BOX1: Twin pair A**				
**#**	**Non-affected Value 1**	**Affected Value 2**	**Difference Q-value**	**Change % up/down**
**1**	17.111921	5.3906255	8.28E-07	-68.50
**2**	24.686842	8.1035590	5.42E-06	-67.17
**3**	49.505867	24.039217	6.76E-05	-50.44
**4**	25.962824	39.875996	0.002803379	53.59
**5**	15.781162	24.678215	1.66E-05	56.38
**6**	47.121210	75.406510	0.001247889	60.03
**7**	33.518060	57.517000	1.30E-04	71.60
**8**	14.864865	26.935987	0.001854299	81.21
**9**	14.107142	27.698416	6.69E-09	96.34
**10**	15.517400	36.594770	0.003385719	135.83
**11**	3.3827498	13.666667	0.001085497	304.01
**12**	1.1363636	23.245615	0.003799252	1945.61
**BOX2: Twin pair B**				
**#**	**Non-affected Value 1**	**Affected Value 2**	**Difference Q-value**	**Change % up/down**
**1**	12.3949585	1.3513515	1.10E-06	-89.10
**2**	14.4585260	2.6777172	3.35E-05	-81.48
**3**	63.4640540	29.532772	1.27E-11	-53.47
**4**	12.5000000	30.833334	0.005804231	146.67
**5**	16.9000000	30.226072	0.002090683	78.85
**6**	24.7163140	44.550762	2.70E-04	80.25
**7**	39.7959180	73.840880	1.20E-07	85.55
**8**	2.25225230	27.405165	1.34E-06	1116.79
**9**	13.8461550	24.765600	0.002160636	78.86
**10**	17.0431160	28.679487	0.002496304	68.28
**11**	5.9829063	30.860060	1.01E-06	415.80
**12**	25.0000000	71.428570	2.86E-04	185.71
**BOX3: Significantly changed DMRs in twin sisters at risk for CVD**				
**#**	**Chr(hg19)**	**Start**	**End**	**Feature**
**1**	1	180,922,916	180,923,692	*AK056657*
**2**	1	41,981,789	41,982,416	*HIVEP3*
**3**	2	8,596,908	8,597,573	
**4**	5	140,186,793	140,187,268	*PCDHA1*
**5**	X	123,093,924	123,094,582	*STAG2*
**6**	16	28,3798,131	28,379,974	*NPIPL1*
**7**	X	153,046,342	153,046,768	*SRPK3*
**8**	1	151,226,666	151,227,238	*PSMD4*
**9**	4	2,048,730	2,049,144	*C4orf48*
**10**	22	29,866,327	29,866,832	*AB020652*
**11**	17	40,259,522	40,259,977	*DHX58*
**12**	14	106,145,949	106,146,803	*IGHE*

**Fig 2:**
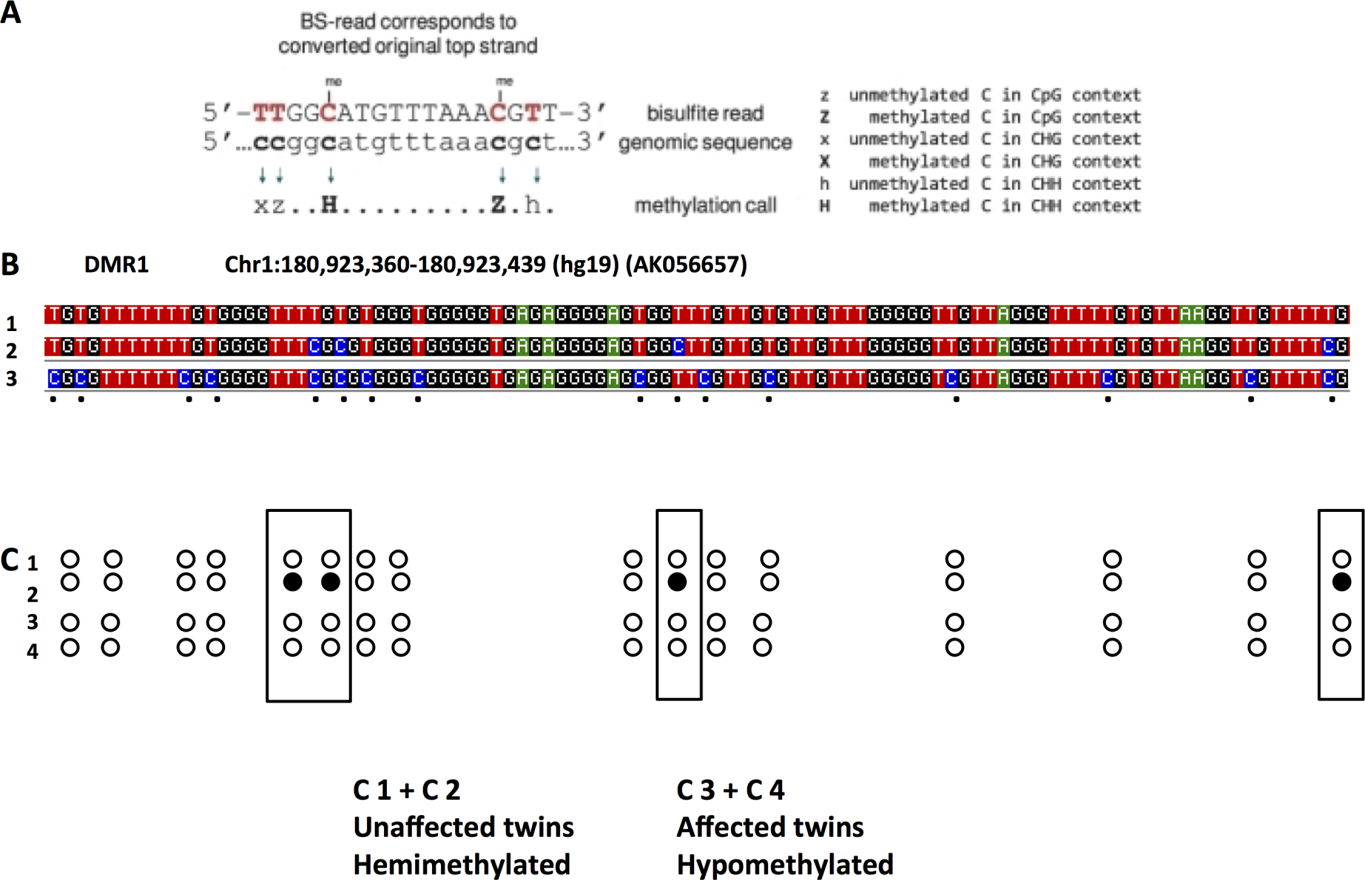
By targeted methyl-C DNA sequencing (principle explained in **A**), DMR1 (see Table 3) was analyzed. Within the 80 bp region analyzed (**B**), 2 patterns were found after bisulfite sequencing. Strands were either completely unmethylated (**B1**) or hemimethylated at 4 sites presenting as heterozygous calls (**B1+B2**). For comparison, the converted upper strand is shown (**B3**). The unaffected twins showed hemimethylation of 4 (boxes) out of 16 methylation sensitive C nucleotides (**C1+C2**) and hypomethylation (loss of methylation) in affected twins (**C3+C4**). Open circle: unmethylated, closed circle: methylated.

By targeted methyl-specific DNA sequencing using DMR1 as target, we were able to sequence 16 methyl-specific C nucleotides in an 80 bp fragment of this DMR. Of these 16 informative nucleotides, 3 CpG sites and 1 CHH site showed hemimethylation (presenting as heterozygotes in the sequence) in the non-affected twin sisters and hypomethylation (presenting as homozygotes in the sequence) in the affected twin sisters **(Fig 2**). This confirmed the loss of methylation in DMR1 in the affected twins.

## Conclusion

The advent of genomics makes it possible to move from hypothesis-driven (mostly unsuccessful) candidate gene studies to hypothesis-generating data-driven genome wide scans. We used this approach to identify the nature and number of the epigenomic changes associated with the increased cardiovascular disease risk in adult women following a pregnancy complicated by pre-eclampsia or their extreme severe phenotypes (HELLP syndrome). We used the best model system available: monozygous parous twin sisters discordant for the extreme phenotype of pre-eclampsia, i.e. HELLP syndrome, assured the best epigenotype-phenotype comparison possible with the guaranteed absence of experimental bias due to polymorphic sequence variation. It also greatly limits the influence of external factors with an effect on the epigenome [12]. The candidate list of 12 DMRs identified in this way will permit targeted screening of DMRs potentially related to CVD risk in large study populations such as the HYPITAT and HYRASS [13,14] and other cohorts [15]. The use of genomic DNA from peripheral blood cells is no limitation. Although counterintuitive, as peripheral blood cells do not represent the cells affected (such as endothelial cells), an increasing number of data show that disease-specific profiles, both at the transcriptional as well as epigenetic levels, of organ-specific diseases are mirrored in the blood, but only accurately identified using genome-wide approaches [16,17]. By conventional methods (serum markers, sonography), there was no identifiable increased risk of CVD at the time of measurement. This could reflect the actual situation with by design (genome-wide analysis) superior informativity generated by methylC-sequencing compared to methods with limited resolution.

Given that DNA methylation provides a lifetime record of environmental exposure [12], the approach we describe therefore could identify a useful source of biomarkers permitting non-invasive presymptomatic risk determination for risk stratification and disease diagnostics allowing early intervention in adult women at risk for CVD after a complicated pregnancy [7].

## Supporting Information

S1 File Approval of Medical board of the VU Medical Center Amsterdam (NL38972.029.12). (PDF)

S2 File Trial registration (NTR5297). (JPG)

S3 File Protocol of the study (protocol). (PDF)

## Abbreviations

CVD: cardiovascular disease
HELLP: Hemolysis, Elevated Liver enzymes, Low Platelets
DMR: differentially methylated region
bp: base pairs; gDNA, genomic DNA
PCR: polymerase chain reaction
