# Supplementary material for: Genome-Wide Identification of Epigenetic Hotspots Potentially Related to Cardiovascular Risk in Adult Women after a Complicated Pregnancy
Source: PLoS One. 2016 Feb 12;11(2):e0148313. doi: 10.1371/journal.pone.0148313 (PMC4752476; doi:10.1371/journal.pone.0148313)
Supplement: S1 File — (PDF) [file pone.0148313.s001.pdf]

De Boelelaan 1117
1081 HV Amsterdam
postbus 7057
1007 MB Amsterdam
telefoon 020 444 4444
www.VUmc.nl

Medisch Ethische Toetsingscommissie
VU medisch centrum
voorzitter: Prof. dr. J.A. Rauwerda
intern postadres: BS7, kamer H-565

mw. prof. dr. C.J.M. de Groot
Afdeling Verloskunde & Gynaecologie
ZH 8F 035

VU medisch centrum

onderwerp
positief oordeel
**NL38972.029.12**

ons kenmerk
2012/316

datum
24 april 2013
doorkiesnummer
(020) 44 45585
e-mail subcom-ethiek.org@vumc.nl

Geachte mevrouw De Groot,

De Medisch Ethische Toetsingscommissie Vrije Universiteit medisch centrum (bevoegd tot oordelen op grond van WMO art. 2.2.a) oordeelt thans in positieve zin omtrent de uitvoering van het onderzoek met titel:

**Early detection of cardiovasculair risk factors after pregnancy complicated by hypertensive disorders**

Aanvrager van het onderzoek: mw. prof. dr. C.J.M. de Groot
Verrichter: VUmc te Amsterdam
METc VUmc registratienummer: 2012/316

**Vergadering en documenten**
De goedkeuring, waartoe in principe besloten is in de plenaire vergadering d.d. 11-10-2012 en de vergaderingen van het dagelijks bestuur d.d. 6-1-2013 en 14-2-2013, is gebaseerd op de volgende documenten:

- Correspondentie tussen METc VUmc en onderzoeker d.d. 1-11-2012, 10-2-2013, 5-3-2013, 18-3-2013 en 15-4-2013
- Begeleidende brief d.d. 24-8-2012
- Akkoordverklaring manager bedrijfsvoering d.d. 2-8-2012
- ABR-formulier versie 2 d.d. 13-3-2013
- Goedkeuring CWO ICaR-VU d.d. 11-11-2011
- Correspondentie voorafgaande aan goedkeuring CWO ICaR-VU
- Risico-classificatie investigator initiated onderzoek
- Protocol zoals ontvangen per e-mail d.d. 10-2-2013
- Patiënteninformatiebrief en toestemmingsverklaring (case) versie 2 d.d. 13-3-2013
- Patiënteninformatiebrief en toestemmingsverklaring (controle) versie 2 d.d. 13-3-2013
- Vragenlijst (case) versie 1 d.d. 8-7-2012
- Vragenlijst (controles) versie 1 d.d. 8-7-2012
- Verzekeringscertificaat WMO proefpersonen Centramed d.d. januari 2013
- Aansprakelijkheidsverzekering Centramed t.b.v. VUmc d.d. januari 2013
- CV onafhankelijk arts (drs. M.E. van Hoorn)
- CV hoofdonderzoeker (prof. dr. C.J.M. de Groot)
- CV uitvoerend onderzoeker (drs. F. van Kesteren)
- Informatiebrief huisarts

**Motivering**
De commissie is van oordeel dat het onderzoek voldoet aan het bepaalde in de van toepassing zijnde wet- en regelgeving, met name de WMO en, voorzover relevant, het ICH/GCP richtsnoer.

**Verzekering**
De METc VUmc heeft vastgesteld dat op correcte wijze uitvoering is gegeven aan de verzekeringsplicht in artikel 7 van de WMO, zoals uitgewerkt in het Besluit verplichte verzekering bij medisch-wetenschappelijk onderzoek met mensen. Naar het oordeel van de commissie gaat het onderzoek gepaard met risico.

**Verplichtingen**
De commissie verwacht dat
- de startdatum van het onderzoek de commissie ter kennis zal worden gebracht
- elke onverwachte bijwerking die zich tijdens het onderzoek voordoet bij de proefpersonen onverwijld aan de commissie gemeld wordt, voorzien van een toelichting betreffende de consequenties voor het onderzoek
- veranderingen in het onderzoeksprotocol aan de commissie worden voorgelegd, voorzien van een toelichting betreffende de consequenties voor de proefpersonen
- jaarlijks een rapport over de voortgang van het onderzoek aan de commissie zal worden toegestuurd
- de beëindiging van het onderzoek, hetzij omdat het onderzoek voltooid is hetzij om andere redenen, de commissie ter kennis zal worden gebracht
- de resultaten van het onderzoek aan de commissie zullen worden gemeld

De commissie heeft de bevoegdheid haar positieve oordeel in te trekken als vaststaat dat de uitvoering van het onderzoek ernstig tekort schiet.
Het voorliggend oordeel verliest zijn geldigheid indien de start van het onderzoek niet binnen 1 jaar plaatsvindt.

**Volledigheidshalve maken wij u er op attent dat het onderzoek pas mag worden uitgevoerd nadat u schriftelijk toestemming hebt gekregen van (het plv. hoofd Instituut Ondersteuning Patiëntenzorg namens) de Raad van Bestuur.**

**Administratief beroep**
Tegen dit besluit kan een belanghebbende op grond van artikel 23 WMO binnen zes weken na de dag waarop het besluit is bekend gemaakt, administratief beroep instellen bij de Centrale Commissie Mensgebonden Onderzoek (CCMO). Het beroepschrift dient u te adresseren aan: CCMO, Postbus 16302, 2500 BH Den Haag.


*Samenstelling commissie*

| | | | |
|---|---|---|---|
| prof. dr. J.A. Rauwerda | voorzitter, chirurg | | |
| dr. K. Hoekman | plv. voorzitter, internist-oncoloog | | |
| mw. M. Baak | verpleegkundige | dr. M. Klein | neuropsycholoog |
| mw. dr. C. Boer | biomedicus | dr. B.W. van Oosten en dr. J. Killestein | neurologen |
| mw. dr. M.A. Bremmer | psychiater | mw. P. Roodenberg | lekenlid |
| dr. E.G. Haarman | kinderlongarts | prof. dr. F.J.H. Tilders | farmacoloog |
| mw. mr. A.J.G.M. Janssen en mr. F.J. Faber | juristen | mw. dr. A.I. Veldkamp en drs. A.J. Wilhelm | ziekenhuisapothekers-klinisch farmacologen |
| dr. M.J.P.A. Janssens en mw. dr. C. Widdershoven | medisch ethici | dr. ir. P. van de Ven en mw. dr. C.B. Terwee | methodologen |
| dr. D. de Jong | chirurg | prof. dr. ir. R. Verdaasdonk en dr. ir. Ing. Th.J.C. Faes | klinisch fysici |


VU medisch centrum

04.001.99 / 1

Met vriendelijke groet,
namens de Medisch Ethische Toetsingscommissie,

prof. dr. J.A. Rauwerda, voorzitter

drs. W.E. van der Voet, secretaris

c.c.: Centrale Commissie Mensgebonden Onderzoek te Den Haag (CCMO) - *digitaal uploaden*
c.c.: f.vankesteren@vumc.nl
c.c.: crb@vumc.nl


*Samenstelling commissie*

| | | | |
|---|---|---|---|
| prof. dr. J.A. Rauwerda | voorzitter, chirurg | | |
| dr. K. Hoekman | plv. voorzitter, internist-oncoloog | | |
| mw. M. Baak | verpleegkundige | dr. M. Klein | neuropsycholoog |
| mw. dr. C. Boer | biomedicus | dr. B.W. van Oosten en dr. J. Killestein | neurologen |
| mw. dr. M.A. Bremmer | psychiater | mw. P. Roodenberg | lekenlid |
| dr. E.G. Haarman | kinderlongarts | prof. dr. F.J.H. Tilders | farmacoloog |
| mw. mr. A.J.G.M. Janssen en mr. F.J. Faber | juristen | mw. dr. A.I. Veldkamp en drs. A.J. Wilhelm | ziekenhuisapothekers-klinisch farmacologen |
| dr. M.J.P.A. Janssens en mw. dr. C. Widdershoven | medisch ethici | dr. ir. P. van de Ven en mw. dr. C.B. Terwee | methodologen |
| dr. D. de Jong | chirurg | prof. dr. ir. R. Verdaasdonk en dr. ir. Ing. Th.J.C. Faes | klinisch fysici |

VU medisch centrum

04.001.99 / 1
