# Supplementary figures and images for: Genome-Wide Identification of Epigenetic Hotspots Potentially Related to Cardiovascular Risk in Adult Women after a Complicated Pregnancy
Source: PLoS One. 2016 Feb 12;11(2):e0148313. doi: 10.1371/journal.pone.0148313 (PMC4752476; doi:10.1371/journal.pone.0148313)

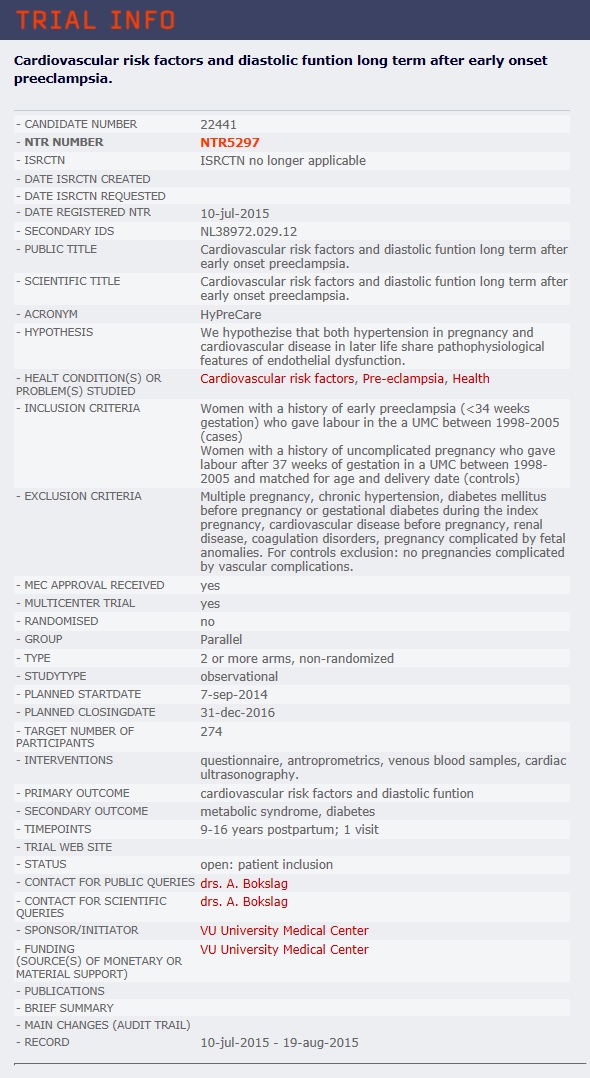

Supplement: S2 File — (JPG) [file pone.0148313.s002.jpg]
