# Supplementary material for: Genome-Wide Identification of Epigenetic Hotspots Potentially Related to Cardiovascular Risk in Adult Women after a Complicated Pregnancy
Source: PLoS One. 2016 Feb 12;11(2):e0148313. doi: 10.1371/journal.pone.0148313 (PMC4752476; doi:10.1371/journal.pone.0148313)
Supplement: S3 File — (PDF) [file pone.0148313.s003.pdf]

Early detection of cardiovascular risk factors after pregnancy complicated by hypertensive disorders

Drs. A. Bokslag	Obstetrics and Gynecology	VUmc
Prof. Dr. C.J.M. de Groot	Obstetrics and Gynecology	VUmc

In cooperation with:

Prof. Dr. W.J. Paulus	Physiology	VUmc
Drs C. Franssen	Physiology	VUmc
Dr. E.H. Serné	Internal Medicine	VUmc
Prof. Dr. Y.M. Smulders	Internal Medicine	VUmc
Prof. Dr. H. Blom	Clinical Chemistry	VUmc
Prof. Dr. H. Niessen	Pathology	VUmc
Prof. Dr D. Boomsma	Psychology	VUmc
Prof. dr C.B. Lambalk	Obstetrics and Gynecology	VUmc
Dr. M. van Dijk	Clinical Chemistry	VUmc
Dr. C.A.R. Lok	Obstetrics and Gynecology	VUmc
prof. Dr. J.W.R. Twisk	Epidemiology and Biostatistics	VUmc

PROTOCOL TITLE: Early detection of cardiovascular risk factors after pregnancy complicated by hypertensive disorders

Protocol ID	**NL38972.029.12**
Short title	Early detection of cardiovascular risk factors after pregnancy
Version	3
Date	16-07-2014
Coordinating investigators l	Prof. dr. C.J.M. de Groot<br>Obstetrics and Gynecology<br>VU University medical centre<br>8F035<br>De Boelelaan 1117<br>1081 HV Amsterdam<br>The Netherlands<br>+31-20-4444851
Principal investigator	Drs. A. Bokslag, Obstetrics and Gynecology, VUmc<br>Tel: 020-4443239.<br>Email: a.bokslag@vumc.nl
Sponsor (in Dutch: verrichter/opdrachtgever)	Prof. dr. C.J.M. de Groot<br>Obstetrics and Gynecology
Independent physician(s)	Drs. M.E. van Hoorn, Obstetrics and Gynecology, VUmc. Tel:020-4442585. Email: m.vanhoorn@vumc.nl
Laboratory sites	VUmc, Clinical Chemistry, De Boelelaan 1117, 1081 HV Amsterdam

PROTOCOL SIGNATURE SHEET

Name	Signature	Date
**For non-commercial research,**<br>**Head of Department:**<br>Prof. dr. C.J.M. de Groot<br>Obstetrics and Gynecology		
**Coordinating Investigator/Project leader**<br>Prof. dr. C.J.M. de Groot<br>Obstetrics and Gynecology		
**Principal Investigator:**<br>Drs. A. Bokslag<br>Obstetrics and Gynecology		

Table of contents

1 Background and main objective page 7

2 Metabolic syndrome and diastolic dysfunction page 16

2.1 Introduction
2.2 Objective
2.3 Study design
2.4 Methods
2.5 Study population
2.5.1 Population
2.5.2 Inclusion criteria
2.5.3 Exclusion criteria
2.5.4 Sample size calculations
2.6 Study parameters/endpoints
2.7 Withdrawal of individual subjects
2.8 Statistical analysis

3 **Microparticles** page 20

3.5 Introduction
3.6 Objective
3.3 Study design
3.4 Methods
3.5 Study population
3.5.1 Population
3.5.2 Inclusion criteria
3.5.3 Exclusion criteria
3.5.4 Sample size calculations
3.6 Study parameters/endpoints
3.7 Withdrawal of individual subjects
3.8 Statistical analysis

4 **Study flow chart** page 23

5 **Safety reporting** page 24

5.5 Section 10 WMO event
5.6 Adverse and serious adverse events
5.7 Follow up of adverse events

6 **Ethical considerations** page 25
6.5 Regulation statement

6.6 Recruitment and consent
6.7 Benefits and risks assessment, group related
6.8 Compensation for injury
6.9 Incentives

7 **Administrative aspects and publication** page 26

7.5 Handling and storage of data and documents
7.6 Amendments
7.7 Annual progress report
7.8 End of study report
7.9 Public disclosure and publication policy

8 **References** page 28

Summary

Background: Cardiovascular disease is the leading cause of death for women in the Western world. Since there is a lack of insight in the pathophysiology, predicting and prevention of cardiovascular disease remains challenging. Hypertensive disorders are a common complication of pregnancy. Epidemiological studies have described an association between hypertensive disorders in pregnancy and the development of cardiovascular disease later in life. Recently, we found that women with previous pregnancies complicated by hypertension have increased modifiable risk factors for cardiovascular disease, years after pregnancy compared to women with previous uncomplicated pregnancies. This implies that pregnancy can potentially be a tool as “stress test” unmasking underlying defects, thus identifying women at increased risk for cardiovascular events at young age.

Hypothesis: We hypothesize that both hypertension in pregnancy and cardiovascular disease in later life share pathophysiological features of cardiac, (micro)vascular and metabolic perturbations.

Aim: To gain insight in the pathophysiology and mechanism of a cardiovascular risk later in life using pregnancy as a stress test. We ultimately plan with these data to develop a discriminating test to predict an individual's cardiovascular risk later in life.

Research Questions: In women with previous preeclampsia and increased cardiovascular risk after their pregnancy we will investigate the hypothesis on two levels including local on tissue level and systemic level:
1. Identification of vascular dysfunction, leading to an increased prevalence of the metabolic syndrome and microparticle levels
2. Identification of diastolic heart dysfunction, one of the earliest signs of cardiac failure, especially in "asymptomatic" women

Methods: We designed an observational cohort study. Women will be invited 9-16 years after they gave labor in the VUmc. The cases consist of women with a history of pregnancy complicated by early preeclampsia (<34 of gestation). The controls are women without any vascular complications of their pregnancy who gave labor around the same time in the VUmc. These women will be screened for cardiovascular risk factors by answering a questionnaire, antroprometrics, venous blood samples, circulating microparticle levels and a cardiac ultrasonography.

Risks and benefits: Risks of participation are very small and associated with venous blood sampling (e.g. hematoma, vasovagal collaps and skin infection). We believe that the scientific gain that this study intends outweighs the discomfort that may occur.

Expected Results: We expect to gain insight in the pathophysiology of the increased cardiovascular risk after preeclampsia and find highly sensitive and specific tests to predict an individual's cardiovascular risk by unravelling the shared pathophysiology. This opens opportunities for prevention of cardiovascular disease at a relatively young age.

1. Background and objective

Cardiovascular disease is the leading cause of death in women in the Western World and a major cause of morbidity. In the Netherlands, heart disease and stroke was the cause of death in almost 30% of the women who died in 2010.(1)
Prediction of an individual's risk for cardiovascular disease is particularly difficult in younger women since diagnostic tools seem to be less sensitive and specific than for men. (2-4) (5;6)
This study focuses on women with a history early preeclampsia during pregnancy. Hypertensive complications of pregnancy are a major cause of maternal en neonatal morbidity and mortality throughout the world (7). The prevalence of preeclampsia is about 4% of all pregnancies. Recent studies have described an association between hypertensive disorders in pregnancy and cardiovascular morbidity and mortality later in life (8-14). The risk of death from cardiovascular disease for women with preeclampsia is 8-fold higher than for women who did not have preeclampsia (15;16), this is confirmed by several population studies and autopsy data (11;15)
It is hypothesized that pregnancy acts as a "stress test" for the mother. Maladaptation of the cardiovascular and metabolic systems in pregnancy might unravel women with a formerly unknown, subclinical impaired organ function. The concept of pregnancy as a "stress test, unmasking underlying defects of the cardiovascular system, opens opportunities, as

hypertensive disorders in pregnancy might identify those women at risk for heart disease and stroke, at a relatively young age. Tailored interventions might help to change the lifestyle and promote healthy ageing of the women at risk. However, these interventions should be based on understanding the pathophysiology of both diseases (hypertension in pregnancy and hypertension later in life). Currently the pathophysiological link is largely unknown and there are several hypotheses about the association between both disorders. It is unclear whether hypertension during pregnancy and cardiovascular disease share common antecedents or that damage from hypertensive disorders during pregnancy leads to cardiovascular disease development later in life. The third hypothesis is a combination of both theories: hypertension during pregnancy and cardiovascular disease has a common cause but the development of hypertension in pregnancy will deteriorate cardiovascular dysfunction. (17) Determinations of risk factors, which discriminate between individuals who will develop cardiovascular disease, are lacking. It is unknown after what time interval following the complicated pregnancy, (subclinical) cardiovascular disorders may become manifest.
Development of a risk profile based on understanding the pathophysiology, will prevent uncoordinated, not evidence based preventive strategies and finally improve healthy maternal aging.

We hypothesize that both hypertension in pregnancy and cardiovascular disease share pathophysiological features of vascular dysfunction. It is known from several other studies that (micro) vascular dysfunction is linked to the metabolic syndrome and is a predictor of cardiovascular disease (10;18-21). Vascular dysfunction causes an increased arterial stiffness, arteriosclerosis and impaired microcirculation. Without intervention this will eventually progress into increased systolic pressure, left ventricular hypertrophy, impaired coronary perfusion and heart failure (22;23). Generalized (micro) vascular dysfunction is an important feature of severe preeclampsia. Several mediators of vascular dysfunction have shown to be up regulated in preeclampsia (7;24).
This study will focus on early identification of signs of cardiovascular disease and individual risk factors by investigating a cohort of women 9 till 16 years following a pregnancy complicated by early preeclampsia (onset <34 weeks of gestational age) as compared to women who have had no vascular complications in pregnancy (controls).
We will focus on the presence of cardiovascular risk factors at tissue and systemic level. The risk factors will be assessed by diastolic heart dysfunction (chapter 2), one of the earliest signs of cardiac failure in asymptomatic women, measuring the presence of the metabolic syndrome and impaired levels of circulating microparticles (chapter 3) .
The time interval of 9-16 years allows us to rule out direct pregnancy related alterations, bias by subsequent pregnancies, and allocates progression of the cardiovascular syndrome into a stage where it is detectable.
It is hypothesized that early preeclampsia, with an onset before 34 weeks of gestation, has a partly different pathophysiology compared to late onset preeclampsia. (25;26) Early onset preeclampsia is associated with more adverse maternal and neonatal outcomes at both short- and long term. (16;26) Epidemiological studies describe the strongest associations between preeclampsia and cardiovascular disease for early onset preeclampsia. (16;27) Therefore this study will focus on women with a history of early onset preeclampsia, before 34 weeks of gestation. This will help elucidate the pathophysiological link between preeclampsia and cardiovascular disease.

In conclusion, hypertensive disorders during pregnancy are related to morbidity and mortality caused by cardiovascular disease in later life. Understanding the pathophysiological link between both disorders allows screening for risk factors after

preeclampsia. Therefore the ultimate aim of our study is to de develop a discriminating test to predict an individual's risk for cardiovascular disease later in life using pregnancy as a stress test. And, hereby create possibilities for early intervention, secondary prevention and long term improvements in outcome.

2. Metabolic syndrome and diastolic dysfunction

2.1 Introduction

The metabolic syndrome defines a cluster of interconnected factors including insulin resistance, hypertension, abdominal obesity and dyslipidemia. The syndrome increases the risk of numerous diseases with high socioeconomic costs including cardiovascular disease. A recent meta-analysis described a 2-fold increase in cardiovascular outcomes, it is suggested that this risk might be even higher in women relative to men (28).
Various studies describe a higher prevalence of the metabolic syndrome in women with a history of hypertension in pregnancy, (21;29-33) suggesting a relationship between the increased risk for the metabolic syndrome after preeclampsia and the development of cardiovascular disease. (34) The studies performed describe a high prevalence of the metabolic syndrome relatively soon after pregnancy (1 to 7 years postpartum) in mostly heterogeneous groups (preeclampsia and gestational hypertension combined) and a mean gestational age of delivery at (near) term. In normal pregnancy the circulation and the metabolism of a woman adapts to meet de physiological demands of a foetus. In women with preeclampsia, such cardiovascular and metabolic changes seem exaggerated.(10;12) It is suggested by Sattar et al. that these cardiovascular and metabolic responses could reveal women at risk for cardiovascular disease later in life. (12)(figure 1)

Diastolic heart failure (DHF), also referred to as heart failure with normal left ventricular ejection fraction (HFNEF), accounts for more than 50% of all heart failure patients. Predisposing conditions for diastolic heart failure are older age, female gender, diabetes and

obesity, arterial hypertension, and left ventricular (LV) hypertrophy (35). Cardiovascular disease is the primary cause of death in women, while in men this is the second cause of death (36). Diastolic dysfunction is one of the first signs of cardiovascular disease (35). It is the corner stone in the diagnosis of heart failure with normal ejection fraction, but it has also a high prevalence in heart failure with reduced ejection fraction.
Although in DHF left ventricular systolic function is preserved, diastolic dysfunction is evident from impaired LV relaxation and increased LV stiffness. The prevalence of HFNEF is rising with 1% per year, thereby rapidly turning it into the most prevalent heart failure phenotype over the next decennium (37).
Studies performed in women during and directly after their pregnancies complicated by preeclampsia show signs of cardiac diastolic dysfunction (38;39). Currently there are no reports of cardiac function with a larger time interval post partum that focussed on diastolic function after a hypertensive complication in pregnancy.

Therefore we will investigate the presence of the metabolic syndrome and we will investigate whether women with a history of early preeclampsia show signs of diastolic left ventricular dysfunction or eventually diastolic heart failure 9 to 16 years after their pregnancy.

2.2 Objective:

To investigate the presence of the metabolic syndrome and diastolic function in women with a history of early preeclampsia (<34 weeks) as compared to controls.

2.3 Study design

In an observational cohort study women with a history of early preeclampsia (<34 weeks) will be compared with normal controls for the presence of cardiovascular risk factors and diastolic cardiac function 9 to 16 years after their pregnancy. The duration of the study will be one year. The study will be performed in the VUmc in Amsterdam.

2.4 Methods

- All patients and controls will be asked to fill in a questionnaire (see attachment: F1)
- Body height, body weight, waist and hip circumference, systolic and diastolic blood pressure will be measured. Pulse pressure and mean arterial pressure will be calculated.
- Venous blood samples will be taken after overnight fasten and tested for: cholesterol, HDL, LDL, triglycerides, free fatty acids, insulin, HbA1c, fasten glucose to assess insulin sensitivity using HOMA, NT-proBNP, hsCRP andproinflammatory cytokines (IL-6, TNF-alpha)Urine (collected in the morning at home) will be tested for microalbuminuria/creatinin ratio.
- An electrocardiogram will be made to exclude atrial fibrillation.
- Cardiac function will be asses by echocardiography (35):
    - Left ventricular ejection fraction (LVEF, biplane Simpson's method)
    - Left ventricular end diastolic volume index (LVEDVI), left ventricular mass index (LVMI).
    - Left atrial volume, left atrial volume indexed to body surface area (LAVI)
    - Tissue Doppler (E/E')
    - Blood flow Doppler (E/A, DT, Ard-Ad)

The criteria for the presence of the metabolic syndrome will be defined according to the to the most widely used definition of the Third report of the National Cholesterol Education program (NCEP: ATP III) (40). To meet the criteria of the NCEP: ATP III it is necessary to have at least 3 or more of the following (41):

- Waist circumference >88 cm,
- Triglyceride levels ≥1.7 mmol/L,
- HDL-cholesterol <1.29 mmol/L
- Blood pressure ≥130/85 mmHg
- Fasting glucose levels ≥5.7mmol/L)

Diastolic dysfunction is defined as the presence of (35):

1. Tissue Doppler (E/E' > 15)

2. If Tissue Doppler yields an *E/E'* ratio of 15 > E/E' > 8, additional non-invasive investigations are required;

$E/A_{>50\,yr} < 0.5$ and $DT_{>50\,yr} > 280$ ms
or
Blood flow Doppler of mitral valve or pulmonary veins (Ard-Ad > 30 ms)
or
Left atrial volume index (LAVI > 40 ml/m^2)
or

Echo measures of LV mass index (LVMI > 122 g/m^2)
or
Electrocardiographic evidence of atrial fibrillation
or
Plasma levels of natriuretic peptides NT-proBNP > 220 pg/ml
or
BNP > 200 pg/ml.

The above is according to the consensus statement on the diagnosis of heart failure with normal left ventricular ejection fraction (35).

Diastolic heart failure is defined as the presence of (35):

1. Signs or symptoms of heart failure (questionnaire, see attachment: F1)
2. Normal or mildly abnormal systolic left ventricular function
(LVEF > 50%, LVEDVI < 97mL/m^2)
3. Evidence of diastolic LV dysfunction (see above).

2.5 Study population

2.5.1 Population

Cases (women who had a pregnancy complicated by preeclampsia): All consecutive women who had a pregnancy complicated by early preeclampsia (before 34 weeks gestation) in the VUmc between 1998 and 2005 will be eligible for the study. Between 1998 and 2005 312 women delivered after early preeclampsia. All cases are coded by date of delivery. Starting from 2005 going back to 1998, all coded cases with preeclampsia will be verified and checked for inclusion criteria as described below. All consecutive eligible cases will be invited by letter of the Head of the department of Obstetrics, Professor CJM de Groot, for participating in this project.
Controls (non-exposed): All women who delivered in the VUmc between 1998 and 2005 after a normotensive pregnancy will be eligible for the study. We will invite women to participate with an uncomplicated pregnancy and delivery within 3 months of the case and match them for ethnicity, parity and maternal age.

2.5.2 Inclusion criteria

Cases (exposed preeclampsia)

- Women with a history of early preeclampsia (<34 weeks gestation)
- Preeclampsia is defined according to the criteria of the ISSHP (42): A systolic blood pressure of ≥ 140 mm Hg and/or a diastolic blood pressure of ≥ 90 mm Hg measured on two occasions at least 4 hours apart, in combination with proteinuria (≥ 300 mg/24 h or 2+ dipstick) developing after 20 weeks of gestation, in a previously normotensive woman.
- Delivery in the VUmcbetween 1998 and2005

Controls (non exposed)

- Women with a history of uncomplicated normotensive pregnancy
- Delivery > 37 weeks gestation
- Delivery in the VUmc between 1998 and 2005

2.5.3 Exclusion criteria

For both patients as controls:

- Multiple pregnancy

- Chronic hypertension before pregnancy
- Diabetes mellitus before pregnancy or gestational diabetes during the index pregnancy
- Cardiovascular disease before pregnancy
- Renal disease
- Coagulation disorders
- History of pregnancy complicated by fetal anomalies
- Raynaud's syndrome
- Any kind of medication
- Currently pregnant
- Pregnancy in the last six months
- Currently breastfeeding

2.5.4 sample size calculation

We are the first to analyze the prevalence of diastolic heart failure in women with a history of early onset preeclampsia years after pregnancy. Therefore our sample size calculation is based on data of the prevalence of diastolic dysfunction in a randomly recruited European population with a mean prevalence of normal diastolic function <40 years of 92.8%. (43) To detect a difference in prevalence of 10%, with a power of 80% and a two sided α of 0.05 we need 137 women in both groups.

2.6 Study parameters/endpoints

The primary outcome measure will be a difference in the presence of the metabolic syndrome, and a difference in diastolic function or appearance of diastolic heart failure between cases and controls. The secondary outcome measure will be a difference in cardiovascular parameters and differences in metabolic and cardiovascular blood measurements (venous blood samples 4.4).

2.7 Withdrawal of individual subjects

Subjects can leave the study at any time for any reason if they wish to do so without any consequences. The investigator can decide to withdraw a subject from the study for urgent medical reasons. In case of withdrawal: venous blood samples will be destroyed and test results will not be used.

2.8 Statistical analysis

The difference in prevalence of the metabolic syndrome, expressed as percentages, between cases and controls, and the difference in diastolic function will be analyzed using unpaired t-tests, Mann-Whitney U tests or Chi-square tests, as appropriate. . Multivariable analysis will be performed for studying the influence of confounding factors including obesity and parity. Multiple logistic regression analysis will be performed to assess the influence of demographic variables, pregnancy characteristics, and metabolic status. A value of $P < 0.05$ is considered statistically significant. All analyses will be analyzed using SPSS20 software (Chicago, IL).

3. Microparticles

3.1 Introduction

Microparticles are small vesicles released from cells by "shedding" upon activation or apoptosis. They range in size from 0.1-1 µm (44). They are involved in many biological processes such as: coagulation, inter cellular communication, cell-survival, inflammation, angiogenesis and endothelial dysfunction. Human blood contains microparticles that originate from various blood cells or endothelial cells. The majority of cell-derived microparticles in plasma originate from platelets. Platelet derived microparticles expose high numbers of binding sites for coagulation factors (45). Evidence is accumulating that microparticles are of pathophysiological relevance in autoimmune, cardiovascular, and thromboembolic diseases and inflammatory disorders.

In normal pregnancy, the concentration of microparticles is decreased at a gestational age of 12 weeks and then gradually increases again to post partum levels. During pregnancy, the placenta is an additional source for microparticles, because syncytiotrophoblast cells release microparticles into the maternal blood. It has been suggested that microparticles modulate several of the key-processes in preeclampsia, including inflammation, coagulation, and platelet activation (44). The concentration of trophoblast-derived microparticles is increased in preeclampsia compared to normal pregnancy. These increased numbers are thought to reflect increased shedding and apoptosis of trophoblast cells due to placental hypoperfusion and subsequent ischemic events (46).

There is no consensus about the changes in microparticle levels in preeclampsia (44). This is due to the differences in patient selection, laboratory techniques and blood sample handling. However, several studies agree on the use of endothelial-derived microparticles as biomarkers for endothelial dysfunction (47-49). It has been shown that microparticles from preeclamptic patients impair vascular dilatation in vitro (46). Endothelial derived microparticles are not only elevated in preeclampsia but also in other vascular disorders like coronary artery disease (50). The interest in microparticles as markers or mediators in cardiovascular disease has grown substantially during the past years. It remains to be elucidated whether microparticles are a cause or a consequence of cardiovascular disease, because infectious agents, cytokines and metabolic disturbances are all shown to affect the release of microparticles (49;51).

Pregnancy and in particular preeclampsia, is associated with hypercoagulability. Rafik et al. investigated women more than one year after delivery and showed that women with a history of preeclampsia show signs of hypercoagulability as indicated by higher thrombin generation and higher platelet derived microparticle levels (45). Whether this increased hypercoagulability and endothelial dysfunction are the cause of the increased cardiovascular disease in formerly preeclamptic patients has not been investigated yet. Therefore, we hypothesize that increased numbers of procoagulant circulating microparticles 9-16 years after an early preeclampsia lead to hypercoagulability coinciding with endothelium dysfunction, and become eventually clinical manifest by cardiovascular disease.

3.2 Objective

To investigate circulating numbers and cellular origin of microparticles in women with a history of early preeclampsia (<34 weeks).

3.3 Study design

In an observational cohort study women with a history of early preeclampsia (<34 weeks) will be compared with women with uncomplicated pregnancies (controls) for the concentration of total circulating microparticles and more specific endothelial-derived

microparticles, 9 to 16 years after their pregnancy. The duration of the study will be one year. The study will be performed in the VUmc in Amsterdam.

3.4 Methods
One blood sample (4.5 ml) will be taken from the antecubital vein without a tourniquet through a 20-gauge needle using a vacutainer system into a 4.5 ml tube containing buffered sodium citrate. Plasma will be isolated and samples will be stored in -80°C freezer until assayed. (52).

3.5 Study population

3.5.1 Population
Cases (women who had a pregnancy complicated by preeclampsia): All consecutive women who had a pregnancy complicated by early preeclampsia (before 34 weeks gestation) in the VUmc between 1998 and 2005 will be eligible for the study. Between 1998 and 2005 312 women delivered after early preeclampsia.
Controls (non-exposed): All women who delivered in het VUmc between 1998 and 2005 after a normotensive pregnancy will be eligible for the study. We will invite women to participate with an uncomplicated pregnancy and delivery within 3 months of the case and match them for ethnicity, parity and maternal age.

3.5.2 Inclusion criteria
Cases (exposed preeclampsia)

- Women with a history of early preeclampsia (<34 weeks gestation)
- Preeclampsia is defined according to the criteria of the ISSHP (42): A systolic blood pressure of ≥ 140 mm Hg and/or a diastolic blood pressure of ≥ 90 mm Hg measured on two occasions at least 4 hours apart, in combination with proteinuria (≥ 300 mg/24 h or 2+ dipstick) developing after 20 weeks of gestation, in a previously normotensive woman.
- Delivery in the VUmc between 1998 and 2005

Controls (non exposed)

- Women with a history of uncomplicated normotensive pregnancy
- Delivery > 37 weeks gestation
- Delivery in the VUmcbetween 1998 and 2005

3.5.3 Exclusion criteria
For both patients as controls:

- Multiple pregnancy
- Chronic hypertension before pregnancy
- Diabetes mellitus before pregnancy or gestational diabetes during the index pregnancy
- Cardiovascular disease before pregnancy
- Renal disease
- Coagulation disorders
- History of pregnancy complicated by fetal anomalies
- Raynaud's syndrome
- Any kind of medication
- Currently pregnant
- Pregnancy in the last six months

- Currently breastfeeding

3.5.4 Sample size calculation

We are the first to investigate microparticle levels in women with a history of early preeclampsia years after the index pregnancy. Six weeks after pregnancy there is a concentration of total microparticles of 7.3 IU (10^9 / L) with a standard deviation of 2 IU (10^9 / L) (52) To detect a difference of means of 20% (1.5 IU) with 80% power and a two sided α of 0.05 we need 28 women in both groups.

3.6 Study parameters/endpoint

The primary outcome will be a difference in circulating number and cellular-origin of microparticles between patients and controls.
Furthermore, correlation with blood pressure and diastolic function will be calculated to investigate whether the concentration of microparticles is an independent predictor of cardiovascular function or disease.

3.7 Withdrawal of individual subjects

Subjects can leave the study at any time for any reason if they wish to do so without any consequences. The investigator can decide to withdraw a subject from the study for urgent medical reasons. In case of withdrawal: venous blood samples will be destroyed and test results will not be used.

3.8 Statistical analysis

The difference in microparticle levels between patients and controls will be analyzed using two-sample t-test, as appropriate.
Multivariate analysis will be performed for confounding factors including obesity and parity. Linear regression analysis will be performed to assess the influence of demographic variables, pregnancy characteristics and metabolic status. A value of $P < 0.05$ is considered statistically significant. All analyses will be analyzed using SPSS 20 software (Chicago, IL)

4. Study flow chart

Prior to the test day a questionnaire will be send to all 137 cases and 137 controls, these will be collected at the testing day. A urine sample will be collected in the morning at home and will be tested for microalbuminuria/creatinin ratio. All women will be asked to retain food and beverages from midnight on.
When arriving at the hospital 5 blood samples of 5 ml will be taken after overnight fast from the antecubital vein without a tourniquet through a 20-gauge needle using a vacutainer system for measuring cholesterol, HDL, LDL, triglycerides, free fatty acids, insulin, HbA1c, fasten glucose,NT- proBNP, hsCRP, proinflammatory cytokines (IL-6, TNF-alpha). In 60 women (30 cases and 30 controls) one more blood sample will be taken for the isolation of microparticles. Then in all women body height, body weight, waist and hip circumference, systolic and diastolic blood pressure will be measured. Afterwards a cardiac ultrasound for assessing diastolic heart function is performed in all women.

**Prior to test day:**	**Questionnaire**
**Test day:**	**Take questionnaire and morning urine sample to the VUmc**
**09:00h**	**Venous blood samples**
**09:15h**	**Breakfast**
**09:30h**	**Anthropometrics, systolic and diastolic blood pressure**
**09:45h**	**Cardiac ultrasound**
**10:15h**	**End of the test day**

5. Safety reporting

5.1 Section 10 WMO event

In accordance to section 10, subsection 1, of the WMO, the investigator will inform the subjects and the reviewing accredited METC if anything occurs, on the basis of which it appears that the disadvantages of participation may be significantly greater than was foreseen in the research proposal. The study will be suspended pending further review by the accredited METC, except insofar as suspension would jeopardise the subjects' health. The investigator will take care that all subjects are kept informed.

5.2 Adverse and serious events

The only adverse events that theoretically could occur are an infection, occurrence of a hematoma and vasovagal collaps related to sampling of venous blood.

Adverse events are defined as any undesirable experience occurring to a subject during the study, whether or not considered related to [the investigational product / the experimental treatment]. All adverse events reported spontaneously by the subject or observed by the investigator or his staff will be recorded.
A serious adverse event is any untoward medical occurrence or effect that at any dose:

- results in death;
- is life threatening (at the time of the event);
- requires hospitalisation or prolongation of existing inpatients' hospitalisation;
- results in persistent or significant disability or incapacity;
- is a congenital anomaly or birth defect;
- is a new event of the trial likely to affect the safety of the subjects, such as an unexpected outcome of an adverse reaction, lack of efficacy of an IMP used for the treatment of a life threatening disease, major safety finding from a newly completed animal study, etc.

All SAEs will be reported through the web portal ToetsingOnline to the accredited METC that approved the protocol, within 15 days after the sponsor has first knowledge of the serious adverse reactions.
SAEs that result in death or are life threatening should be reported expedited. The expedited reporting will occur not later than 7 days after the responsible investigator has first knowledge of the adverse reaction. This is for a preliminary report with another 8 days for completion of the report.

5.3 Follow-up of adverse events

All adverse events will be followed until they have abated, or until a stable situation has been reached. Depending on the event, follow up may require additional tests or medical procedures as indicated, and/or referral to the general physician or a medical specialist.

6. ETHICAL CONSIDERATIONS

6.1 Regulation statement

The study will be conducted according to the principles of the Declaration of Helsinki (Adopted by the 18th WMA General Assembly, Helsinki, Finland, June 1964, and amended by the: 59th WMA General Assembly, Seoul, October 2008) and in accordance with the Medical Research Involving Human Subjects Act (WMO).

6.2 Recruitment and consent

The principal investigator of the Department of Gynecology will contact, inform and eventually recruit the women, 9-16 years postpartum. The women will be provided with written information and have to fill out a form to give consent for the anthropometry, venous blood samplingand the cardiac ultrasound. All women have at least 1 month time to consider their decision to participate in this study.

6.3 Benefits and risks assessment, group relatedness

Cardiovascular disease is the most important cause of death for women in the Western World, therefore there is a public interest for studies concerning cardiovascular disease in women, with the ultimate aim to reduce risks for women. In our study women who participate contribute to an increase of knowledge and hereby they contribute to the public health. The study will give us insight in specific cardiovascular risk factors and the risk status of women with a pregnancy complicated by preeclampsia 9-16 years after their index pregnancy. The personal advantage of being informed of the risk status for cardiovascular disease, outweighs the disadvantages of venapunction, since this will provide options for (secondary) prevention, if needed. If women have modifiable risk factors and agree to inform the family physician, they have the opportunity for a tailored made secondary prevention program coordinated by their family physician based on the NHG standard Cardiovascular Risk Management (53). Previous studies have shown that women with a history of complicated pregnancy are extremely motivated to participate in a study evaluating their complicated pregnancy years after their event. They experience this as extra care for themselves and not as stressful nor exciting (54).

6.4 Compensation for injury

The investigators have an insurance which is in accordance with the legal requirements in the Netherlands (Article 7 WMO and the Measure regarding Compulsory Insurance for Clinical Research in Humans of 23rd June 2003). This insurance provides cover for damage to research subjects through injury or death caused by the study.
€ 450.000, -- (i.e. four hundred and fifty thousand Euro) for death or injury for each subject who participates in the Research;
€ 3.500.000, -- (i.e. three million five hundred thousand Euro) for death or injury for all subjects who participate in the Research;
€ 5.000.000,-- (i.e. five million Euro) for the total damage incurred by the organisation for all damage disclosed by scientific research for the Sponsor as 'verrichter' in the meaning of said Act in each year of insurance coverage.
The insurance applies to the damage that becomes apparent during the study or within 4 years after the end of the study.

6.5 Incentives

Subjects will receive a compensation for their travel expenses.

7. Administrative aspects and publication

7.1 Handling and storage of data and documents

We will collect data from the pregnancy, the birth of the child and the period after delivery in a standard procedure from the medical file of the woman, data already collected during normal healthcare. Additional information we will collect by questionnaire about current cardiovascular risk factors, lab results (blood and urine)and a cardiac ultrasound. The extra information will only be collected after informed consent of the women. We will ask consent for every item (see patient information letter and informed consent)

A part of the (lab) measurements will be done immediately, the microparticles will be isolated after we have collected all the blood samples of the amount of women we need for this part of the study. The remaining blood and/or urine will be stored for future scientific research. This blood will be coded and depersonalised with a unique number based on the sequence of blood collection. The key for this number will be safeguarded by the investigator and a confidential committee at the department of Obstetrics and Gynecology. Women will be asked explicitly permission to store the remaining blood for 15 years.
The storage of data will take place in a central database with two separated parts. The first part will consist of personal information about the participants. This part is only accessible for members of the scientific commission and will be separated from the research results. Research number, name, address en contact information will be filed. The members of the scientific commission will check if the participants signed and returned the informed consent paper. This part will not contain research results and will not be accessible for the investigators. The second part consists of research results with specific codes for each participant and is only accessible for members of the scientific commission. The keys of the codes will be only accessible by the investigator and the confidential committee of the department of Obstetrics and Gynecology. The scientific commission is responsible for the quality and safety of the information stored in the database.

7.2 Amendments

Amendments are changes made to the research after a favourable opinion by the accredited METC has been given. All amendments will be notified to the METC that gave a favourable opinion. Non-substantial amendments will not be notified to the accredited METC and the competent authority, but will be recorded and filed by the sponsor.

7.3 Annual progress report

The sponsor/investigator will submit a summary of the progress of the trial to the accredited METC once a year. Information will be provided on the date of inclusion of the first subject, numbers of subjects included and numbers of subjects that have completed the trial, serious adverse events/ serious adverse reactions, other problems, and amendments.

7.4 End of study report

The investigator will notify the accredited METC of the end of the study within a period of 8 weeks. The end of the study is defined as the last patient's last visit.
In case the study is ended prematurely, the investigator will notify the accredited METC, including the reasons for the premature termination.
Within one year after the end of the study, the investigator/sponsor will submit a final study report with the results of the study, including any publications/abstracts of the study, to the accredited METC.

7.5 Public disclosure and publication policy

Not applicable

8. Reference List

Reference List

(1) http://statline.cbs.nl/StatWeb/publication/?DM=SLNL&PA=7052_95&D1=a&D2=1-2&D3=0&D4=39-41&HDR=G2,G1&STB=G3,T&VW=T. 21-12-2011.
Ref Type: Online Source

(2) Stramba-Badiale M, Fox KM, Priori SG, Collins P, Daly C, Graham I, et al. Cardiovascular diseases in women: a statement from the policy conference of the European Society of Cardiology. Eur Heart J 2006 Apr;27(8):994-1005.

(3) Douglas PS, Ginsburg GS. The evaluation of chest pain in women. N Engl J Med 1996 May 16;334(20):1311-5.

(4) Stramba-Badiale M, Fox KM, Priori SG, Collins P, Daly C, Graham I, et al. Cardiovascular diseases in women: a statement from the policy conference of the European Society of Cardiology. Eur Heart J 2006 Apr;27(8):994-1005.

(5) Vaccarino V, Badimon L, Corti R, de WC, Dorobantu M, Hall A, et al. Ischaemic heart disease in women: are there sex differences in pathophysiology and risk factors? Position paper from the working group on coronary pathophysiology and microcirculation of the European Society of Cardiology. Cardiovasc Res 2011 Apr 1;90(1):9-17.

(6) Sullivan AK, Holdright DR, Wright CA, Sparrow JL, Cunningham D, Fox KM. Chest pain in women: clinical, investigative, and prognostic features. BMJ 1994 Apr 2;308(6933):883-6.

(7) Grill S, Rusterholz C, Zanetti-Dallenbach R, Tercanli S, Holzgreve W, Hahn S, et al. Potential markers of preeclampsia--a review. Reprod Biol Endocrinol 2009;7:70.

(8) Craici I, Wagner S, Garovic VD. Preeclampsia and future cardiovascular risk: formal risk factor or failed stress test? Ther Adv Cardiovasc Dis 2008 Aug;2(4):249-59.

(9) Roberts JM, Hubel CA. Pregnancy: a screening test for later life cardiovascular disease. Womens Health Issues 2010 Sep;20(5):304-7.

(10) Rodie VA, Freeman DJ, Sattar N, Greer IA. Pre-eclampsia and cardiovascular disease: metabolic syndrome of pregnancy? Atherosclerosis 2004 Aug;175(2):189-202.

(11) Romundstad PR, Magnussen EB, Smith GD, Vatten LJ. Hypertension in pregnancy and later cardiovascular risk: common antecedents? Circulation 2010 Aug 10;122(6):579-84.

(12) Sattar N, Greer IA. Pregnancy complications and maternal cardiovascular risk: opportunities for intervention and screening? BMJ 2002 Jul 20;325(7356):157-60.

(13) Yinon Y, Kingdom JC, Odutayo A, Moineddin R, Drewlo S, Lai V, et al. Vascular dysfunction in women with a history of preeclampsia and intrauterine growth restriction: insights into future vascular risk. Circulation 2010 Nov 2;122(18):1846-53.

(14) Smith GC, Pell JP, Walsh D. Pregnancy complications and maternal risk of ischaemic heart disease: a retrospective cohort study of 129,290 births. Lancet 2001 Jun 23;357(9273):2002-6.

(15) Irgens HU, Reisaeter L, Irgens LM, Lie RT. Long term mortality of mothers and fathers after pre-eclampsia: population based cohort study. BMJ 2001 Nov 24;323(7323):1213-7.

(16) Bellamy L, Casas JP, Hingorani AD, Williams DJ. Pre-eclampsia and risk of cardiovascular disease and cancer in later life: systematic review and meta-analysis. BMJ 2007 Nov 10;335(7627):974.

(17) Roberts JM, Gammill H. Pre-eclampsia and cardiovascular disease in later life. Lancet 2005 Sep 17;366(9490):961-2.

(18) Alberti KG, Eckel RH, Grundy SM, Zimmet PZ, Cleeman JI, Donato KA, et al. Harmonizing the metabolic syndrome: a joint interim statement of the International Diabetes Federation Task Force on Epidemiology and Prevention; National Heart, Lung, and Blood Institute; American Heart Association; World Heart Federation; International Atherosclerosis Society; and International Association for the Study of Obesity. Circulation 2009 Oct 20;120(16):1640-5.

(19) Isomaa B, Almgren P, Tuomi T, Forsen B, Lahti K, Nissen M, et al. Cardiovascular morbidity and mortality associated with the metabolic syndrome. Diabetes Care 2001 Apr;24(4):683-9.

(20) Serne EH, de Jongh RT, Eringa EC, IJzerman RG, Stehouwer CD. Microvascular dysfunction: a potential pathophysiological role in the metabolic syndrome. Hypertension 2007 Jul;50(1):204-11.

(21) Forest JC, Girouard J, Masse J, Moutquin JM, Kharfi A, Ness RB, et al. Early occurrence of metabolic syndrome after hypertension in pregnancy. Obstet Gynecol 2005 Jun;105(6):1373-80.

(22) Borlaug BA, Kass DA. Ventricular-vascular interaction in heart failure. Heart Fail Clin 2008 Jan;4(1):23-36.

(23) Kass DA. Ventricular arterial stiffening: integrating the pathophysiology. Hypertension 2005 Jul;46(1):185-93.

(24) Young BC, Levine RJ, Karumanchi SA. Pathogenesis of preeclampsia. Annu Rev Pathol 2010;5:173-92.

(25) Huppertz B. Placental origins of preeclampsia: challenging the current hypothesis. Hypertension 2008 Apr;51(4):970-5.

(26) von DP, Magee LA, Roberts JM. Subclassification of preeclampsia. Hypertens Pregnancy 2003;22(2):143-8.

(27) Mongraw-Chaffin ML, Cirillo PM, Cohn BA. Preeclampsia and cardiovascular disease death: prospective evidence from the child health and development studies cohort. Hypertension 2010 Jul;56(1):166-71.

(28) Mottillo S, Filion KB, Genest J, Joseph L, Pilote L, Poirier P, et al. The metabolic syndrome and cardiovascular risk a systematic review and meta-analysis. J Am Coll Cardiol 2010 Sep 28;56(14):1113-32.

(29) Hermes W, Franx A, van Pampus MG, Bloemenkamp KW, van der Post JA, Porath M, et al. Classic Cardiovascular Risk Factors in Women who had Hypertensive Disorders in Late Pregnancy. 2011.
Ref Type: Unpublished Work

(30) Berends AL, De Groot CJ, Sijbrands EJ, Sie MP, Benneheij SH, Pal R, et al. Shared constitutional risks for maternal vascular-related pregnancy complications and future cardiovascular disease. Hypertension 2008 Apr;51(4):1034-41.

(31) Smith GN, Walker MC, Liu A, Wen SW, Swansburg M, Ramshaw H, et al. A history of preeclampsia identifies women who have underlying cardiovascular risk factors. Am J Obstet Gynecol 2009 Jan;200(1):58.

(32) Barden AE, Beilin LJ, Ritchie J, Walters BN, Michael C. Does a predisposition to the metabolic syndrome sensitize women to develop pre-eclampsia? J Hypertens 1999 Sep;17(9):1307-15.

(33) Pouta A, Hartikainen AL, Sovio U, Gissler M, Laitinen J, McCarthy MI, et al. Manifestations of metabolic syndrome after hypertensive pregnancy. Hypertension 2004 Apr;43(4):825-31.

(34) Girouard J, Giguere Y, Moutquin JM, Forest JC. Previous hypertensive disease of pregnancy is associated with alterations of markers of insulin resistance. Hypertension 2007 May;49(5):1056-62.

(35) Paulus WJ, Tschope C, Sanderson JE, Rusconi C, Flachskampf FA, Rademakers FE, et al. How to diagnose diastolic heart failure: a consensus statement on the diagnosis of heart failure with normal left ventricular ejection fraction by the Heart Failure and Echocardiography Associations of the European Society of Cardiology. Eur Heart J 2007 Oct;28(20):2539-50.

(36) Sterfte naar hoofdgroepen van doodsoorzaken, RIVM. 2007. http:, www.nationaalkompas.nl/gezondheid-en-ziekte/sterfte-levensverwachting-en-daly-s/sterfte-naar-doodsoorzaak/waaraan-overlijden-mensen-in-nederland/, editors. 2011.
Ref Type: Online Source

(37) Owan TE, Hodge DO, Herges RM, Jacobsen SJ, Roger VL, Redfield MM. Trends in prevalence and outcome of heart failure with preserved ejection fraction. N Engl J Med 2006 Jul 20;355(3):251-9.

(38) Zandstra M, Stekkinger E, van der Vlugt MJ, van Dijk AP, Lotgering FK, Spaanderman ME. Cardiac diastolic dysfunction and metabolic syndrome in young women after placental syndrome. Obstet Gynecol 2010 Jan;115(1):101-8.

(39) Melchiorre K, Sutherland GR, Baltabaeva A, Liberati M, Thilaganathan B. Maternal cardiac dysfunction and remodeling in women with preeclampsia at term. Hypertension 2011 Jan;57(1):85-93.

(40) Kassi E, Pervanidou P, Kaltsas G, Chrousos G. Metabolic syndrome: definitions and controversies. BMC Med 2011;9:48.

(41) Third Report of the National Cholesterol Education Program (NCEP) Expert Panel on Detection, Evaluation, and Treatment of High Blood Cholesterol in Adults (Adult Treatment Panel III) final report. Circulation 2002 Dec 17;106(25):3143-421.

(42) Brown MA, Lindheimer MD, de SM, Van AA, Moutquin JM. The classification and diagnosis of the hypertensive disorders of pregnancy: statement from the International Society for the Study of Hypertension in Pregnancy (ISSHP). Hypertens Pregnancy 2001;20(1):IX-XIV.

(43) Kloch-Badelek M, Kuznetsova T, Sakiewicz W, Tikhonoff V, Ryabikov A, Gonzalez A, et al. Prevalence of left ventricular diastolic dysfunction in European populations based on cross-validated diagnostic thresholds. Cardiovasc Ultrasound 2012;10:10.

(44) Toth B, Lok CA, Boing A, Diamant M, van der Post JA, Friese K, et al. Microparticles and exosomes: impact on normal and complicated pregnancy. Am J Reprod Immunol 2007 Nov;58(5):389-402.

(45) Rafik HR, Curvers J, Berntorp E, Eriksson M, Bremme K. Increased thrombin generation in women with a history of preeclampsia. Thromb Res 2009 Feb;123(4):580-6.

(46) van der Post JA, Lok CA, Boer K, Sturk A, Sargent IL, Nieuwland R. The functions of microparticles in pre-eclampsia. Semin Thromb Hemost 2011 Mar;37(2):146-52.

(47) Nozaki T, Sugiyama S, Sugamura K, Ohba K, Matsuzawa Y, Konishi M, et al. Prognostic value of endothelial microparticles in patients with heart failure. Eur J Heart Fail 2010 Nov;12(11):1223-8.

(48) Lekakis J, Abraham P, Balbarini A, Blann A, Boulanger CM, Cockcroft J, et al. Methods for evaluating endothelial function: a position statement from the European Society of Cardiology Working Group on Peripheral Circulation. Eur J Cardiovasc Prev Rehabil 2011 Mar 11.

(49) Tushuizen ME, Diamant M, Sturk A, Nieuwland R. Cell-derived microparticles in the pathogenesis of cardiovascular disease: friend or foe? Arterioscler Thromb Vasc Biol 2011 Jan;31(1):4-9.

(50) Gonzalez-Quintero VH, Jimenez JJ, Jy W, Mauro LM, Hortman L, O'Sullivan MJ, et al. Elevated plasma endothelial microparticles in preeclampsia. Am J Obstet Gynecol 2003 Aug;189(2):589-93.

(51) Diamant M, Tushuizen ME, Sturk A, Nieuwland R. Cellular microparticles: new players in the field of vascular disease? Eur J Clin Invest 2004 Jun;34(6):392-401.

(52) Lok CA, van der Post JA, Sargent IL, Hau CM, Sturk A, Boer K, et al. Changes in microparicle numbers and cellular origin during pregnancy and preeclampsia. Hypertens Pregnancy. In press 2011.

(53) http://nhg.artsennet.nl/kenniscentrum/k_richtlijnen/k_nhgstandaarden/Samenvattingskaartje-NHGStandaard/M84_svk.htm. 2011.
Ref Type: Online Source

(54) Gaugler-Senden IP, Duivenvoorden HJ, Filius A, De Groot CJ, Steegers EA, Passchier J. Maternal psychosocial outcome after early onset preeclampsia and preterm birth. J Matern Fetal Neonatal Med 2011 May 11.
